# Effect of endurance training and PGC-1α overexpression on calculated lactate production volume during exercise based on blood lactate concentration

**DOI:** 10.1038/s41598-022-05593-1

**Published:** 2022-01-31

**Authors:** Reo Takeda, Yudai Nonaka, Katsuyuki Kakinoki, Shinji Miura, Yutaka Kano, Daisuke Hoshino

**Affiliations:** 1 Bioscience and Technology Program, Department of Engineering Science, The University of Electro-Communications, 1-5-1 Chofugaoka, Tokyo 182-8585 Chofu, Japan; 2 Institute of Liberal Arts and Science, Kanazawa University, Ishikawa, Japan; 3 Blue Wych Limited Company, Kanagawa, Japan; 4 Laboratory of Nutritional Biochemistry, Graduate School of Nutritional and Environmental Sciences, University of Shizuoka, Shizuoka, Japan

**Keywords:** Metabolism, Biochemistry

## Abstract

Lactate production is an important clue for understanding metabolic and signal responses to exercise but its measurement is difficult. Therefore, this study aimed (1) to develop a method of calculating lactate production volume during exercise based on blood lactate concentration and compare the effects between endurance exercise training (EX) and PGC-1α overexpression (OE), (2) to elucidate which proteins and enzymes contribute to changes in lactate production due to EX and muscle PGC-1α OE, and (3) to elucidate the relationship between lactate production volume and signaling phosphorylations involved in mitochondrial biogenesis. EX and PGC-1α OE decreased muscle lactate production volume at the absolute same-intensity exercise, but only PGC-1α OE increased lactate production volume at the relative same-intensity exercise. Multiple linear regression revealed that phosphofructokinase, monocarboxylate transporter (MCT)1, MCT4, and citrate synthase equally contribute to the lactate production volume at high-intensity exercise within physiological adaptations, such as EX, not PGC-1α OE. We found that an exercise intensity-dependent increase in the lactate production volume was associated with a decrease in glycogen concentration and an increase in P-AMPK/T-AMPK. This suggested that the calculated lactate production volume was appropriate and reflected metabolic and signal responses but further modifications are needed for the translation to humans.

## Introduction

Lactate, which is produced in the glycolytic pathway, was previously considered as a cause of fatigue that inhibits prolonged muscle contraction. However, lactate is now widely recognized as an efficient energy source used in systemic organs^1^. Monocarboxylate transporters (MCTs) play an important role in the use of lactate as an energy source^2^. Lactate in the blood is taken up by cells of various tissues via MCTs, where it is converted to pyruvate and utilized in the mitochondria for oxidative phosphorylation^3,4^. This concept, known as the lactate shuttle theory, was proposed by Dr. Brooks^4,5^.

Exercise accelerates lactate production in working muscles, especially during a high-intensity exercise and at the onset of exercise. The reason is that during such exercise, glycogen degradation and glycolytic flux are enhanced by glycogenolytic and glycolytic enzymes, such as glycogen phosphorylases and phosphofructokinase (PFK) in the muscles. Pyruvate produced in the activated glycolysis pathway has two major fates: pyruvate is taken up by mitochondria and converted to acetyl-coenzyme A by pyruvate dehydrogenase (PDH), or it is converted to lactate by lactate dehydrogenase (LDH) in the cytosol. Since LDH is originally a high active enzyme^6^, most pyruvate is converted to lactate because the enzymatic activity of LDH is significantly higher than that of PDH at the higher intensity^7^ (more than 65% VO_2max_). Indeed, the pyruvate and lactate concentrations are in equilibrium at around 1:100 for exercised skeletal muscles at more than 75% VO_2max_ in humans^8^ and at 70% VO_2max_ in dogs^9^.

Blood lactate concentration during exercise decreases at the same absolute intensity in rats after endurance exercise training^10,11^ and in mice with skeletal muscle-specific peroxisome proliferator-activated receptor gamma coactivator 1-alpha (PGC-1α) overexpression^12–14^. The reason is that the increase in muscle mitochondrial density due to endurance exercise training prevents the increase in the concentrations of Pi^15^ and ADP^16^, which are activators of glycolytic enzyme activity and flux of glycogenolysis. Similarly, mice with PGC-1α overexpression have rich muscle mitochondrial density^13,14^. However, endurance exercise training and PGC-1α overexpression affect not only the mitochondrial volume but also MCTs and the glycolytic enzyme protein contents in the skeletal muscles^12–14^. In these situations, it is unclear which proteins and enzymes involved in lactate metabolism contribute to the suppression of muscle lactate production during exercise. Since it has been suggested that the adaptation of lactate metabolism after endurance training would be different between relative and absolute exercise intensities^10^, it is necessary to examine the relationship between these proteins and lactate production for each relative and absolute exercise intensities.

Recently, it was suggested that lactate is an important factor for inducing mitochondrial biogenesis. Lactate increases PGC-1α mRNA expression and mitochondrial biogenesis in cultured muscle cells^17^ and mouse skeletal muscles^18^. An exercise-induced increase in PGC-1α mRNA was observed at above the lactate threshold, but not below it^19^. In addition, when exercise was performed with suppressed muscle lactate production and accumulation by dichloroacetate administration in mice, mitochondrial quantitative adaptation by chronic exercise training was attenuated^20^. These previous studies suggest that lactate production in the skeletal muscle during exercise may be important for exercise-induced mitochondrial biogenesis. Mitochondrial biogenesis is induced by the activation of important kinases, such as AMP-activated protein kinase (AMPK), Ca^2+^/calmodulin-dependent protein kinase (CaMK) II, and p38 mitogen-activated protein kinase (MAPK)^21^. An increase in lactate production volume during exercise may reflect the activations of signaling molecules involved with mitochondrial biogenesis, yet the relationship between lactate production volume and their activations requires further elucidation.

Lactate production is an important clue for understanding the metabolic responses and adaptation to exercise. However, the measurement of the lactate production volume requires the use of isotope tracer techniques^22–24^ and/or muscle biopsy^16,25^, which are highly invasive. To reduce the application of invasive treatments, we considered a method of calculating the lactate production volume based on blood lactate concentration, which is commonly used as an index of exercise intensity. The blood lactate concentrations during the increment exercise test determine three phases and two thresholds (1st: 40–60% VO_2max_, 2nd: 60–90% VO_2max_), which are closely related to physiological and respiratory parameters^26^. Since blood lactate concentration is a balance between lactate production and uptake in organs, if lactate uptake can be determined, lactate production can be calculated based on blood lactate concentration. Previous studies also calculated the volume of lactate production based on blood lactate concentration following supramaximal exercise^27–29^ using the model developed by Freund et al.^30^. However, this method can only determine the lactate production volume during recovery following supramaximal exercise. We can resolve this problem by improving the experimental design and calculation method, such as by determining the lactate uptake capacity prior to the incremental exercise test. Lactate uptake capacity can be calculated on the basis of the dynamics of blood lactate concentration after lactate administration. It is practically useful to establish a simple equation consisting of a few parameters and acquired data to estimate the lactate production volume. Therefore, we set the following three purposes of this study: (1) to establish a simple method for calculating the lactate production volume during exercise based on blood lactate concentration and compare the effects between endurance exercise training and PGC-1α overexpression on lactate production volume, (2) to elucidate which proteins and enzymes contribute to the changes in lactate production due to endurance exercise training and muscle PGC-1α overexpression, and (3) to elucidate the relationship between the calculated lactate production volume and signaling molecule phosphorylations of mitochondrial biogenesis.

## Materials and methods

### Animals

In the endurance exercise training experiments, 5-week-old male C57BL6/J mice were obtained from Japan SLC Inc. (Shizuoka, Japan). Transgenic mice with PGC-1α-b overexpression were generated using the method described below. All mice were housed in a room with a temperature of 23 °C ± 2 °C with a 12-h light/dark cycle and were allowed food and water ad libitum. All experiments were approved by the Institutional Animal Care and Use Committee of the University of Electro-Communications (Approval No. 33 and No. 19001; Tokyo, Japan) and followed the ARRIVE guidelines. All methods were performed in accordance with the relevant guidelines and regulations.

### Endurance exercise training

After all mice were acclimatized to the treadmill (MELQUEST, Toyama, Japan) exercise for 10 m/min × 5 min and 20 m/min × 5 min for 3 days, the mice were randomly divided into a control group (CON; n = 7) and a 6-week endurance exercise training group (EX; n = 7). The EX group was subjected to endurance exercise training on a treadmill for 30 min a day, five times a week, for a total of 6 weeks. The treadmill speed during training was 20 m/min for 30 min in the first week, 20 m/min for 10 min in the second week, and 25 m/min for 20 min in the third week. Thereafter, EX was set to 25 m/min for 30 min. The treadmill incline was 0°. The mice were run using an air blow when the mice came down to the back of the belt. The body weight was measured in both groups before performing all exercises.

### PGC-1α transgenic mice

We used muscle-specific PGC-1α overexpression mice (PGC-1α OE) to clarify the changes in lactate uptake capacity and production volume in mice with increased lactate uptake. Based on previous reports, PGC-1α overexpression induced high levels of MCT1 protein in skeletal muscle^12^ and lactate uptake rates^31^. PGC-1α OE were generated using the previously described methods^32–34^. Briefly, the human skeletal muscle actin promoter provided by Drs. E. C. Hardeman and K. L. Guven (Children’s Medical Research Institute, Westmead, Australia) was used to generate the PGC-1α-b isoform in the skeletal muscle. Five PGC-1α OE and five WT littermates, all 10-week-old, were used. Furthermore, we revealed the unique phenotypes of this animal, such as high slow muscle fiber composition, mitochondrial mass and capillary density in skeletal muscle, and high maximal oxygen uptake^13,31^.

### Lactate tolerance test

Lactate solution (1.0 mg/g sodium lactate of body weight) was administered intraperitoneally in the awake resting state. The blood lactate concentration was measured from the tail vein before and at 5, 10, 15, 20, 30, 45, and 60 min after lactate injection using a portable blood lactate analyzer (Lactate Pro 2, Arkray, Kyoto, Japan).

### Incremental exercise test

Three hours after the lactate tolerance test, the blood lactate concentration was confirmed to have returned to the resting level, and an incremental exercise test of 2-min run and 2-min rest was performed. The exercise intensity was increased to 10, 20, 25, 30, 35, and 40 m/min. The blood lactate concentration was measured from the tail vein during the rest between exercises with different intensities.

### Tissue sample collection

Twenty-four hours after the incremental exercise test, the mice were sacrificed under anesthesia using isoflurane. The gastrocnemius, soleus, and plantaris muscles were harvested from both legs. All tissues were immediately frozen in liquid nitrogen and stored at − 80 °C.

### Western blot analysis

The lateral gastrocnemius and the whole soleus and plantaris muscles were homogenized in cold RIPA lysis buffer (0.5 M Tris–HCl, 1.5 M NaCl, 2.5% deoxycholic acid, 10% NP-40, 10 mM EDTA, pH 7.4) containing protease inhibitor cocktail (Sigma-Aldrich, St. Louis, MO) and phosphatase inhibitors (PhosSTOP; Roche, Basel, Switzerland). Homogenized samples were centrifuged at 1500*g* for 15 min at 4 °C, and the supernatants were collected. Total protein concentration was quantified using BCA Protein Assay Kit (Pierce, Rockford, IL). Specific protein contents were measured using a Western blot analysis, as described previously^35,36^. An equal amount of protein (10 µg) was loaded onto SDS-PAGE gels and separated. Proteins were transferred to polyvinylidene difluoride membranes by wet transfer (100 V, 80 min). The membranes were blocked with 5% skim milk or 5% bovine serum albumin (BSA) diluted in Tris-buffered saline containing 0.1% Tween-20 (TBS-T) for 1 h at room temperature. The membranes were incubated overnight at 4 °C with primary antibodies diluted 1:1000 in TBS-T containing 5% skim milk or 5% BSA (MCT1 [gift from Dr. H. Hatta, The University of Tokyo, Japan], MCT4 [gift from Dr. H. Hatta], PFK [#55028-1-AP, Proteintech Japan, Tokyo, Japan], and PGC-1α [AB3242, Merck Millipore, Burlington, MA]). After a 1-h incubation with secondary antibody diluted 1:3000 in TBS-T (anti-mouse IgG, NA931, or anti-rabbit IgG, NA9340; Cytiva, Marlborough, MA), the bands were visualized by a chemiluminescence reagent (Nacalai Tesque, Kyoto, Japan) and quantified by densitometry (LAS-4000, Fujifilm, Tokyo, Japan). Ponceau S staining was performed to verify equal loading of samples.

### Mitochondrial enzyme activity

The maximal activities of citrate synthase (CS) were determined in muscle homogenates. In brief, the superficial gastrocnemius and whole soleus and plantaris muscles were homogenized in 100 (vol/wt) of 100-mmol/L potassium phosphate buffer. Maximal activities were quantified using a microplate reader, as described previously^37^.

### Calculation of lactate uptake capacity

The lactate uptake capacity was calculated based on the blood lactate concentration obtained during the lactate tolerance test. Based on reaction kinetics, Eq. (1) was formulated to fit the graphical form of the experimental data of the time series, with blood lactate concentration as the rate-limiting factor^38^. The lactate uptake capacity can be evaluated by the value of the reaction constant “k” of the lactate uptake reaction.

1 $$\frac{{{dC}}_{blood}}{{dt}}= {-{k}\left[\text{Lactate}\right]}^{{n}}$$

Using the time course data from the peak to 60 min, “k” and “n” were calculated to minimize the error between the experimental and estimated values using MATLAB (version R2018b, MathWorks, Natick, MA). The residual sum of squares (RSS) was employed to evaluate the error. RSS was calculated using the following equation:

2 $$\text{RSS}={\sum }_{\text{ j}=1}^{\text{J}}{\upvarepsilon }_{\text{j}}^{2}$$

where ε denotes the residual between the experimental and estimated values. We fixed “n” at the average values of the four groups and recalculated “k,” the index of lactate uptake capacity. To verify whether the calculated “k” represents the lactate uptake capacity, we simulated the decay of blood lactate concentration from 15 mmol/L using the average “k” values of each group in MATLAB.

### Calculation of lactate production volume

The lactate production volume were calculated based on the blood lactate concentration of the incremental exercise test and lactate uptake capacity. The following equation was formulated to calculate lactate production (3):

3 $$\frac{{dC}_{production}}{dt}=\frac{{dC}_{blood}}{dt}+\frac{{dC}_{uptake}}{dt}$$

Lactate production volume was calculated during a 2-min exercise at each intensity. This calculation was based on a blood volume of 72 mL/kg in mice^39^. In order to investigate the lactate production at the same relative exercise intensity, a simulation was performed to calculate the lactate production volume by fixing the blood lactate concentration at 4 mM in Eq. (3).

### Multiple linear regression

Multiple linear regression (MLR) was performed with lactate production as the response variable and PFK, MCT1, and MCT4 proteins and CS activity as explanatory variables using MATLAB. The weights of the four explanatory variables of Eq. (4) were t-valued to quantify their effects on lactate production; b in Eq. (4) is the intercept.

4 $$\left[\text{Lactate production}\right]=\left[\text{PFK}\right]{x}_{1}+\left[\text{MCT}1\right]{x}_{2}+\left[\text{MCT}4\right]{x}_{3}+\left[\text{CS}\right]{x}_{4}+b$$

In order to confirm that the multiple regression analysis was performed correctly, the variance inflation factor (VIF) was calculated as a measure of multicollinearity. The following equation was formulated to calculate VIF (5). r means correlation coefficient.

5 $$\text{VIF} = \frac{1}{1-{r}^{2}}$$

When the value of VIF was 10 or more, multicollinearity was defined.

### Signaling phosphorylations and glycogen concentration

We investigated the relationship between the calculated lactate production volume and signaling molecule phosphorylations involved in mitochondrial biogenesis at various exercise intensities. A total of 30 male C57BL6/J mice (11 weeks old) were acclimatized for 2 days and then acclimatized to treadmill exercise (10 m/min × 5 min and 20 m/min × 5 min) for 3 days. Subsequently, they performed interval exercise, specifically 2 min of running and 2 min of rest × 10 times at 0, 10, 20, 30, or 40 m/min. The mice were performed cervical dislocation immediately after the exercise and the gastrocnemius muscles were harvested in both legs. All tissues were immediately frozen in liquid nitrogen and stored at − 80 °C. We measured phosphorylated (p-) AMPKα [Thr172, #2535, Cell Signaling Technology (CST), Danvers, MA], AMPKα [#5831, CST], p-p38 MAPK [Thr180/Tyr182, #9211, CST], p38 MAPK [#9212, CST], p-CAMKII [Thr286, #12716, CST], and CaMKII [#4436, CST]), which are signaling molecules known to regulate mitochondrial biogenesis^21^, using Western blot analysis. In addition, to quantify glycogen concentrations after exercise at various intensities, the gastrocnemius muscle of another leg was homogenized with 0.3 M perchloric acid. After acid hydrolysis and neutralized, glucose concentration was assayed using the enzymatic methods (716251, Roche, Basel, Switzerland).

### Statistical analysis

All data are expressed as mean ± standard error. Two-way analysis of variance was conducted for the lactate tolerance test (training or PGC-1α overexpression × time) and incremental exercise test (training or PGC-1α overexpression × exercise intensity). When an interaction was observed, Sidak’s multiple comparisons test was employed as the post hoc test. The results of lactate uptake capacity, lactate production volume, muscle glycogen concentration, and signaling phosphorylations were examined via one-way analysis of variance for exercise intensity, and Tukey–Kramer’s multiple comparisons test was used as the post hoc test. In addition, the protein levels and CS activity were compared between the CON and EX groups or WT and PGC-1α OE groups using an unpaired t-test. Pearson’s correlation coefficient was employed for the correlation analysis. Statistical significance was set to P < 0.05. Statistical processing without MLR was performed using GraphPad Prism 8 (version 8, GraphPad Software, La Jolla, CA).

## Results

### Effects of endurance exercise training and PGC-1α overexpression on lactate uptake capacity

**Figure 1:**
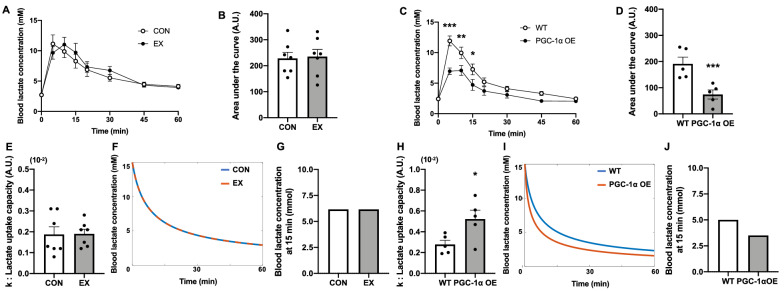
Effects of endurance exercise training and PGC-1α overexpression on lactate uptake capacity. Time course and area under the curve of blood lactate concentration during lactate tolerance test after 6 weeks of endurance exercise training (**A**, **B**) and skeletal muscle-specific PGC-1α overexpression (**C**, **D**). The values of k, which indicate lactate uptake capacity (**E**, **H**). Simulation of blood lactate concentration decay from 15 mM using the mean values of k in each group (**F**, **I**) and simulated blood lactate concentration at 15 min (**G**, **J**). Data are expressed as mean ± SEM. n = 7 in the CON and EX groups. n = 5 in the WT and PGC-1α OE groups. *P < 0.05, **P < 0.01, ***P < 0.001.

The body and muscle weights were not significantly different between the CON and EX groups or between the WT and PGC-1α OE groups (Table 1). During the lactate tolerance test, the blood lactate concentration dynamics and their area under the curves (AUCs) demonstrated no significant difference between the CON and EX groups (Fig. 1A,B). The blood lactate concentrations at 5, 10, and 15 min and AUCs following lactate injection in the PGC-1α OE group were significantly lower than those in the WT group (Fig. 1C,D). Next, we calculated k such that Eq. (1) fit the decay from the peak of blood lactate concentration after the lactate injection, as presented in Fig. S1A. The k values were not significantly different between the CON and EX groups (Fig. 1E). We simulated the change in blood lactate concentration from 15 mM using the mean k value of each group, and the decay in blood lactate concentration from 15 mM did not change (Fig. 1F,G). In contrast, the k values of the PGC-1α OE group were significantly higher than those of the WT group (Fig. 1H). The decay of the blood lactate concentration in the PGC-1α OE group happened faster than that in the WT group (Fig. 1I,J). Indeed, the larger the k value, the faster the decay in blood lactate concentration (Fig. S1B).

**Table 1:** Body and muscle weights.

	CON	EX	WT	PGC-1α OE
Initial body weight (g)	23.0 ± 0.4	23.1 ± 0.4	–	–
Final body weight (g)	28.0 ± 0.6	26.6 ± 0.6	21.8 ± 0.9	22.9 ± 1.3
Gas muscle weight (mg)	111.6 ± 2.9	109.1 ± 2.2	98.2 ± 1.8	99.0 ± 2.1
sol muscle weight (mg)	10.1 ± 0.6	9.6 ± 0.2	9.0 ± 0.4	9.6 ± 0.4
pla muscle weight (mg)	17.9 ± 0.7	16.5 ± 0.5	14.4 ± 1.1	14.0 ± 0.9

Data are expressed as mean ± SEM. n = 7 in the CON and EX groups. n = 5 in the WT and PGC-1α OE groups.

*gas* gastrocnemius, *sol* soleus, *pla* plantaris.

### Effects of endurance exercise training and PGC-1α overexpression on lactate production volume during exercise

**Figure 2:**
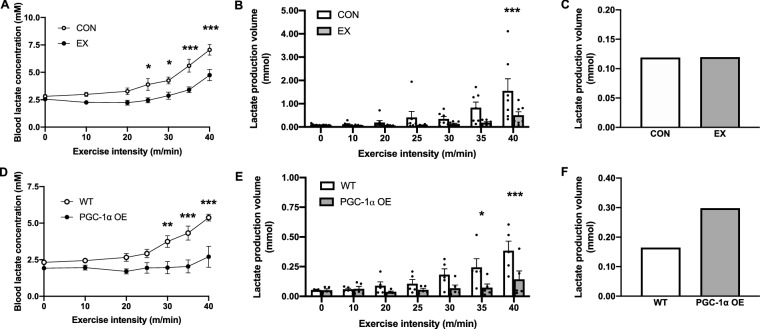
Effects of endurance exercise training and PGC-1α overexpression on lactate production volume. Blood lactate concentrations of CON and EX or WT and PGC-1α OE at each exercise intensity during incremental exercise test (**A**, **D**). The lactate production volume during a 2-min exercise calculated using Eq. (2) utilizing the experimental data (**B**, **E**). Simulated lactate production volume in response to 4 mM blood lactate concentration in each group (**C**, **F**). Data are expressed as mean ± SEM. n = 7 in the CON and EX groups. n = 5 in the WT and PGC-1α OE groups. *P < 0.05, **P < 0.01, ***P < 0.001.

The blood lactate concentration in the EX group was significantly lower than that in the CON group above 25 m/min (Fig. 2A). The calculated lactate production volume in the EX group was significantly lower than that in the CON group at 40 m/min (Fig. 2B). To measure the lactate production volume at the relative same intensity exercise, the lactate production volume at 4 mM of blood lactate concentration was calculated for each group. The lactate production volume was not different between the CON and EX groups (Fig. 2C). However, the blood lactate concentration in the PGC-1α OE group was significantly lower than that in the WT group above 30 m/min (Fig. 2D). The calculated lactate production volume in the PGC-1α OE group was significantly lower than that in the WT group at 35 and 40 m/min (Fig. 2E). The calculated lactate production volume in the PGC-1α OE group at 4 mM was approximately two times higher than that in the WT group (Fig. 2F).

### Effect of endurance exercise training and PGC-1α overexpression on MCTs and PFK proteins and CS activity

**Figure 3:**
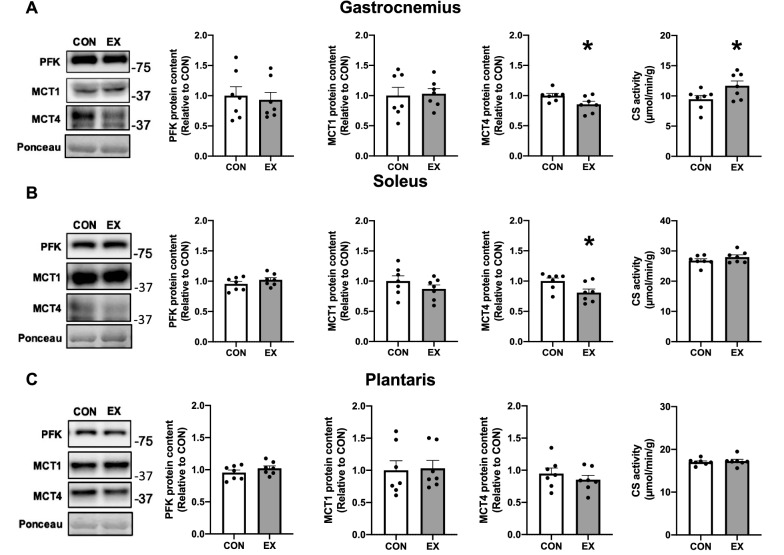
Effect of 6-week endurance exercise training on the PFK and MCT proteins and CS activity in the gastrocnemius (**A**), soleus (**B**), and plantaris (**C**) muscles. Data are expressed as mean ± SEM. n = 7 in each group. *P < 0.05.

The PFK and MCT1 protein contents were not significantly changed in any muscles after a 6-week endurance exercise training (Fig. 3A–C). The MCT4 protein contents were significantly lower in the gastrocnemius and soleus muscles (Fig. 3A,B) but not in the plantaris muscle (Fig. 3C). The CS activities in the gastrocnemius muscle were significantly higher after a 6-week endurance exercise training (Fig. 3A) but not in the soleus and plantaris muscles (Fig. 3B,C).

**Figure 4:**
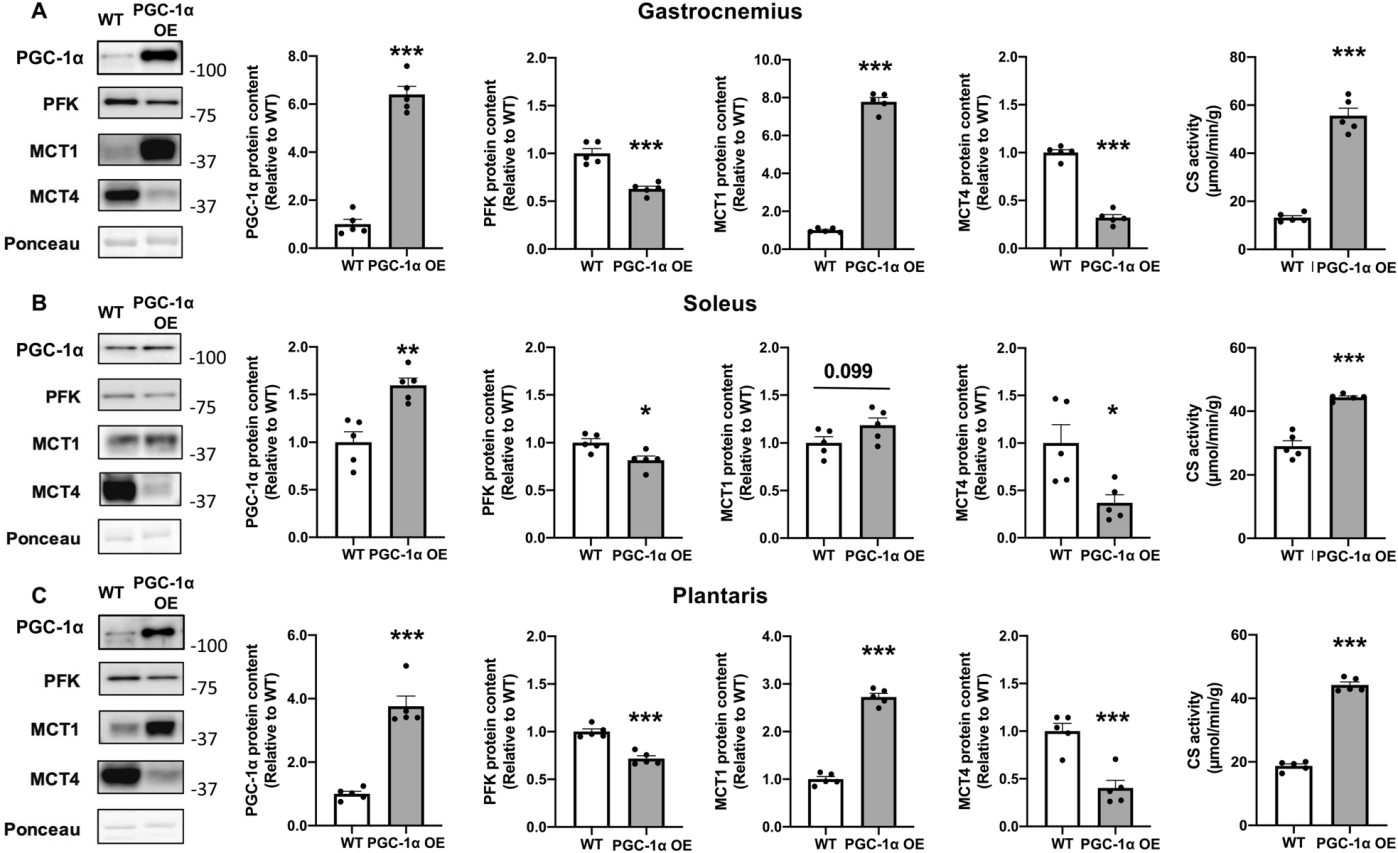
Effect of skeletal muscle-specific PGC-1α overexpression on the PGC-1α, PFK, and MCT proteins and CS activity in the gastrocnemius (**A**), soleus (**B**), and plantaris (**C**) muscles. Data are expressed as mean ± SEM. n = 5 in each group. *P < 0.05, **P < 0.01, ***P < 0.001.

The PFK and MCT4 protein contents were significantly lower in all muscles due to PGC-1α overexpression (Fig. 4A–C). The MCT1 protein contents were significantly higher in the gastrocnemius and plantaris muscles due to PGC-1α overexpression (Fig. 4A,C). Contrarily, the MCT1 protein contents in the soleus muscle tended to be higher due to PGC-1α overexpression (Fig. 4B). The CS activities were significantly higher in all muscles due to PGC-1α overexpression (Fig. 4A–C).

### Multiple linear regression

**Figure 5:**
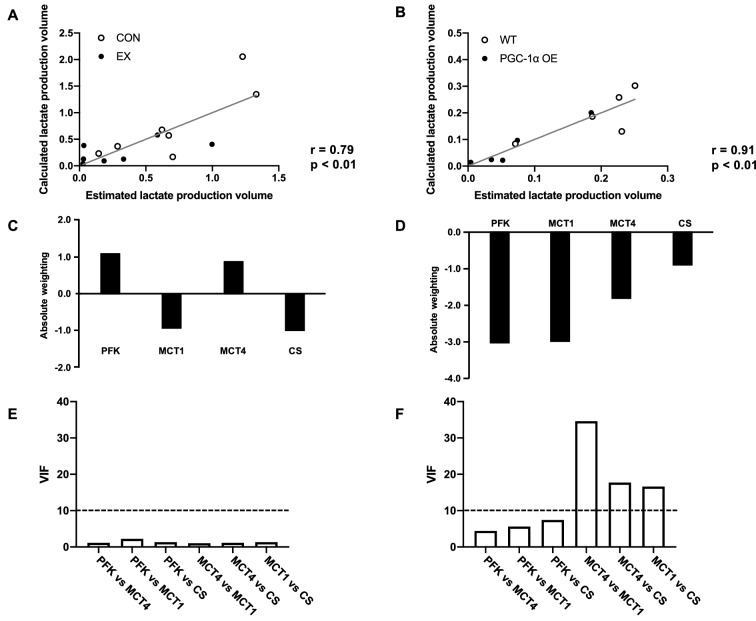
Relationship between lactate production volume and proteins involved in lactate metabolism. The multiple linear regression model of lactate production volume with PFK and MCT proteins and CS activity in CON and EX (**A**) and WT and PGC-1α OE (**B**). Absolute positive or negative weighting of each variable in determining lactate production volume in CON and EX (**C**) and WT and PGC-1α OE (**D**). VIF between variables in CON and EX (**E**) and WT and PGC-1α OE (**F**).

The lactate production estimated from the measured lactate metabolism protein contents and CS activity was significantly positively correlated with the lactate production calculated from the blood lactate concentration (Fig. 5A; r = 0.79, Fig. 5B r = 0.91). When the weighting was verified, the calculated lactate production at 40 m/min was positively influenced by the PFK (+ 1.09) and MCT4 (+ 0.87) protein levels and negatively influenced by the MCT1 protein levels (− 0.94) and CS activity (− 1.00) in the CON and EX groups (Fig. 5C). Contrarily, all parameters had negative effects in the WT and PGC-1α OE groups (Fig. 5D). In particular, the PFK protein level had the strongest effect (− 3.04), followed by the MCT1 (− 3.00) and MCT4 (− 1.83) protein levels. There were several pairs with VIF value of more than 10 in the WT and PGC-1α OE groups, but not in the CON and EX groups (Fig. 5E,F). Multicollinearity was observed in the multiple regression analysis for the WT and PGC-1α OE groups.

### Relationship between calculated lactate production and signaling molecules involved in mitochondrial biogenesis

**Figure 6:**
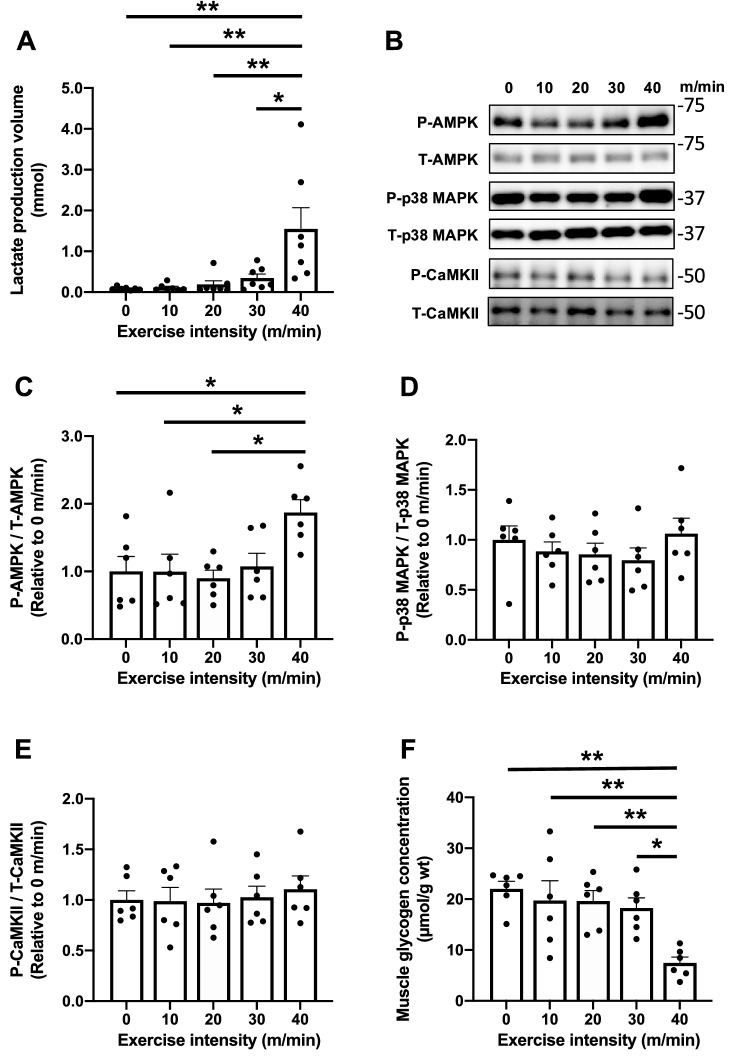
Acute response to various exercise intensities. Lactate production volume (**A**), representative images of signaling molecules (**B**), AMPK (**C**), p38 MAPK (**D**), and CaMKII (**E**) immediately after each exercise. Muscle glycogen concentrations (**F**) measured immediately after each exercise. Data are expressed as mean ± SEM. n = 6 in each group. *P < 0.05, **P < 0.01.

Lactate production volume at an exercise intensity of 40 m/min was significantly higher than those at 0–30 m/min (Fig. 6A). The phosphorylation of AMPK in the 40 m/min group was significantly higher than that in the 0–20 m/min group (Fig. 6B,C). Neither p38 MAPK nor CaMKII changed the phosphorylation state of these kinases (Fig. 6B,D,E). The muscle glycogen concentration measured immediately after the interval exercise at 40 m/min was significantly lower than that at 0–30 m/min (Fig. 6F). These results indicated that exercise intensity-dependent changes in P-AMPK/T-AMPK and muscle glycogen concentration were similar to those in lactate production volume.

## Discussion

We developed a simple method of calculating lactate production volume during exercise by determining the lactate uptake capacity prior to the incremental exercise test. Interestingly, EX and PGC-1α OE decreased lactate production volume at the absolute same-intensity exercise, but only PGC-1α OE increased it at the relative same-intensity exercise, which is at 4 mM of blood lactate concentration. These different results are associated with the changes in the MCT1 protein contents. MLR revealed that PFK, MCT1, and MCT4 and CS activity equally contributed to the regulation of lactate production volume at the absolute high-intensity exercise. Exercise intensity-dependent changes in P-AMPK/T-AMPK and muscle glycogen concentration were similar to those in lactate production volume. These results suggest that the calculated lactate production volume was appropriate and reflected metabolic responses and activation of signaling molecules. Nonetheless, further modification of the proposed method is necessary for applying this method to humans.

In this study, we calculated the lactate uptake capacity before calculating lactate production volume. The “k” in Eq. (1) was used as an index of lactate uptake capacity. Although the k did not change with the 6-week endurance exercise training, it increased with the overexpression of PGC-1α. The MCT1 protein content did not change with the exercise training, but it increased in the gastrocnemius, plantaris, and soleus muscles due to PGC-1α overexpression. Although lactate uptake was not measured directly, high lactate uptake capacity in PGC-1α OE was supported by a previous study, indicating that PGC-1α transfection induced high MCT1 protein content and lactate uptake^31^. CS activity, which is an index of mitochondrial volume, also increased in the gastrocnemius, plantaris, and soleus muscles due to PGC-1α overexpression. The MCT1 protein content and CS activity in the working muscles have positive correlations with the lactate removal rate^28,29^, which was calculated using the model developed by Freud et al.^30^, after maximal exercise in humans. Furthermore, our simulations demonstrated that the rate of blood lactate concentration decay from 15 mM depended on the k values. These experimental data and simulations demonstrate that the lactate uptake capacity calculated from changes in blood lactate concentration after lactate injection is appropriate.

To the best of our knowledge, this is the first study to calculate lactate production volume based on the blood lactate concentration at each exercise intensity by determining the lactate uptake capacity prior to the exercise test. We consider that the lactate production volume calculated using our method was appropriate for the following reasons. First, the calculated lactate production volume increased along with the decreases in muscle glycogen concentration according to the exercise intensity. Since lactate is produced by muscle glycogen degradation, the relationship between lactate production volume and glycogen concentration was reasonable. Second, the values of lactate production volume were within the range of values determined in previous studies using isotope tracer techniques^10,22^ and biopsies^25^. Our findings demonstrated an average value of 27.4 mmol/kg/min in the CON group at 40 m/min, which was calculated to the same unit used in their study^10^. This was actually higher than that in previous studies, in which the lactate production volume at a high-intensity exercise was around 0.5 mmol/kg/min in rats^10^ and humans^22^. Contrarily, the average value was 0.77 mmol/mouse/min in the CON group at 40 m/min. This value was lower than the previous result, which was around 5 mmol/leg/min during 10 min of 70% VO_2_peak exercise^25^. Although our values are not identical to those of the previous studies, they are within the range of those results^10,25^. The difference in these results may be due to the use of different animal species, exercise protocols and/or estimated blood volume per body weight.

Both the endurance exercise training and PGC-1α overexpression significantly reduced the blood lactate concentrations during a high-intensity exercise. These results were consistent with those of previous studies^10–12,14^. At the same time, the endurance exercise training and PGC-1α overexpression decreased the lactate production volume. These results indicate that the decrease in blood lactate concentration at the absolute same-intensity exercise was associated with the decrease in lactate production volume during exercise. This decrease in lactate production and accumulation in the skeletal muscles during exercise were also observed in previous studies using endurance-trained rats^11,15^ and mice with PGC-1α overexpression^14^. Contrarily, Donovan and Brooks reported that the decrease in blood lactate concentration after endurance exercise training was due to the increase in lactate clearance in rats^10^. This different interpretation was considered to be the result of the use of different exercise intensity settings (relative or absolute intensity). Indeed, the lactate turnover (production and removal) rate was reduced at the absolute same-intensity exercise, but the lactate leg utilization (oxidation) was increased at the relative same-intensity exercise after endurance exercise training^23^. Furthermore, in the study by Donovan et al., the lactate turnover rate at relative exercise intensities defined by the blood lactate concentration was higher in the endurance-trained rats than in the control rats and peaked at around 4 mM of the blood lactate concentration^10^. In our study, the lactate production volume at relative exercise intensities was calculated by simulating the lactate production volume when the blood lactate concentration was 4 mM. Indeed, PGC-1α OE did not reach 4 mM and a fixed threshold does not represent the individual threshold response^40^. However, we need to use the blood lactate concentration as an indicator of exercise intensity for comparison with the previous study^10^. The 4 mM is widely used as an indicator of exercise intensity for onset of blood lactate^41,42^. This intensity was considered to be more than 80% VO_2max_ and at the second threshold of the three phase concept^26^. As a result, PGC-1α overexpression, but not endurance exercise training, increased the lactate production volume at the relative same-intensity exercise (4 mM of blood lactate concentration). We speculate that the reason the lactate production volume at the relative same-intensity exercise did not increase after endurance exercise training was that MCT1, which plays a role of lactate uptake^43,44^, did not increase after the endurance exercise training in this study. Stanley et al. found that subjects with higher lactate uptake (lactate disappearance) in response to the same blood lactate concentration have higher lactate production (lactate appearance)^22^. Endurance exercise training usually increases the MCT1 protein contents and lactate uptake rates in the muscles of humans^23,45^ and rodents^43,46^. Therefore, increases in MCT1 in the active muscles would be required to increase the lactate production and uptake volumes during the relative same-intensity exercise.

To elucidate which proteins and enzymes contribute to the changes in lactate production volume at 40 m/min due to endurance exercise training and PGC-1α overexpression, we performed MLR. In the CON and EX groups, the PFK and MCT4 proteins positively contributed whereas the MCT1 protein and CS activity negatively contributed to the regulation of lactate production volume. PFK, MCT1, MCT4, and CS equally contributed to lactate production volume at 40 m/min, suggesting that the regulation of lactate production is complex. The positive effects of PFK and MCT4 are consistent with the results of a previous study, which found that PFK and MCT4 activated glycolytic flux in vitro^47^. However, the correlation coefficient was 0.79, suggesting that other physiological factors also were associated with the regulation for lactate production after training adaptation. Conversely, in the WT and PGC-1α OE groups, all parameters, particularly the PFK and MCT1 protein contents, had a negative effect on lactate production volume. We consider that this analysis was not successful because there was multicollinearity for the analysis with two potential reasons. First, the data distribution was not sufficient for a successful MLR as the blood lactate concentration at the intensity was low in the PGC-1α OE group. In fact, the contribution of MCT4 to lactate production volume was significantly reduced when MLR was performed for low-intensity exercise (10 m/min) in the CON and EX groups (data not shown). Second, other physiological factors increased by exercise training may contribute to the regulation of lactate production, such as capillary density^48^ and proteins related to fat metabolism^49^. Indeed, PGC-1α overexpression is associated with a high capillary density and protein contents involved in fat metabolism^50,51^. Therefore, within physiological adaptations, such as endurance exercise training, PFK, MCTs, and CS equally contribute to the regulation of lactate production volume at a high-intensity exercise.

Finally, we investigated the relationship between lactate production and phosphorylation of signaling molecules of mitochondrial biogenesis or muscle glycogen concentration at different exercise intensities. It was found that lactate production volume and P-AMPK/T-AMPK at an exercise intensity of 40 m/min were significantly higher compared with those at other intensities. This is supported by previous studies that reported exercise intensity-dependent AMPK activation^52,53^. The increase in P-AMPK/T-AMPK is associated with ATP/AMP, ATP/ADP, muscle glycogen levels, and other factors^54^. In fact, the muscle glycogen concentration also exhibited a significantly low value at 40 m/min, in which the lactate production volume exhibited a significantly high in this study. This result also confirmed that the calculated lactate production volume was appropriate because lactate is produced by enhancing glycogen degradation. These results indicate that blood lactate concentration can be used not only as an index of exercise intensity but also as a tool for estimating changes in the metabolic environment and activation of signaling molecules in the working muscles by calculating lactate production volume using the method presented in this study. Therefore, this study expanded the significance of blood lactate concentration measurement.

This study has three limitations. The first pertains to the measurement of lactate uptake capacity at rest because it is known that muscle contraction increased the net lactate uptake when the blood lactate concentration was the same^55^. It has been reported that active recovery promoted a decrease in blood lactate concentration after high-intensity exercise in humans^56^. This suggested that the lactate uptake capacity during the exercise was underestimated in this study. Further studies need to determine the lactate uptake capacity during exercise by injecting lactate before the exercise and measuring the blood lactate concentration for increased accuracy. The second is that we cannot detect lactate production volume increases without changes in blood lactate concentration because the lactate production volume was calculated based on the blood lactate concentration. The lactate shuttle between the muscle fibers and within the cell cannot be considered in this study. It has been reported that the lactate production volume increases without changing the blood lactate concentration^57^. In our study, the lactate production volume may be underestimated, especially at low exercise intensity. Furthermore, mitochondrial pyruvate oxidation increases with the increase in lactate production and exercise intensity^7^. Developing a mathematical model that includes lactate shuttles between fibers and/or within the muscle cell can solve this problem. The third limitation of this study was the use of estimated blood volume per body weight for the calculation of lactate production volume. If the blood volume per body weight largely differs between subjects (e.g., child vs aged and obese vs non-obese), it would affect the results. When considering the application of this method in humans, it would be appropriate to compare between subjects who are of similar age and body composition.

## Conclusion

We established a simple method for calculating the lactate production volume during exercise based on the blood lactate concentration. Endurance exercise training and PGC-1α overexpression decreased the lactate production volume during exercise at the absolute high-intensity exercise. However, at the relative same-intensity exercise, PGC-1α overexpression, but not endurance exercise training, increased lactate production volume during the exercise. These different results would be associated with the changes in the MCT1 protein contents. As results of MLR, within physiological adaptations, such as endurance exercise training, PFK, MCT1, MCT4, and CS equally contribute to the regulation of lactate production during a high-intensity exercise. Finally, the increase in lactate production volume was associated with the increase in P-AMPK/T-AMPK. We confirmed that the calculated lactate production volume was appropriate because it increased with the decrease in glycogen concentration according to the exercise intensity. Nonetheless, further modification is needed for applying this method to humans.

## Supplementary Information

Supplementary Information.
