# Supplementary material for: Effect of endurance training and PGC-1α overexpression on calculated lactate production volume during exercise based on blood lactate concentration
Source: Sci Rep. 2022 Jan 31;12:1635. doi: 10.1038/s41598-022-05593-1 (PMC8803982; doi:10.1038/s41598-022-05593-1)

Supplementary Appendix

Effect of endurance training and PGC-1α overexpression on calculated lactate production volume during exercise based on blood lactate concentration

Reo Takeda[1], Yudai Nonaka[1,2], Katsuyuki Kakinoki[3], Shinji Miura[4], Yutaka Kano[1], Daisuke Hoshino[1,*]

[1]Bioscience and Technology Program, Department of Engineering Science, The University of Electro-Communications, 1-5-1 Chofugaoka, Chofu, Tokyo 182-8585, Japan.

[2] Institute of Liberal Arts and Science, Kanazawa University, Ishikawa, Japan.

[3] Blue Wych Limited Company, Kanagawa, Japan.

[4] Laboratory of Nutritional Biochemistry, Graduate School of Nutritional and Environmental Sciences, University of Shizuoka, Shizuoka, Japan.

*Corresponding Author. Daisuke Hoshino, PhD. Bioscience and Technology Program, Department of Engineering Science, The University of Electro-Communications, 1-5-1 Chofugaoka, Chofu, Tokyo 182-8585, Japan. Tel 81-42-443-5589, Fax 81-42-443-5590, Email: dhoshino@uec.ac.jp

This supplementary appendix was created by authors to provide the validity of calculated k and all original gel/blot images for review.

Fig. S1A is a representative example showing that Eq. *(1)* with k fits the experimental data.
Fig. S1B is the simulation result showing that k represents the lactate uptake capacity.
Fig. S2 is the original gel/blots shown in figure 3 of the main manuscript.
Fig. S3 is the original gel/blots shown in figure 4 of the main manuscript.
Fig. S4 is the original gel/blots shown in figure 6 of the main manuscript.
Fig. S5 is representative Ponceau stains for each sample

Fig. S1. The validity of calculated k

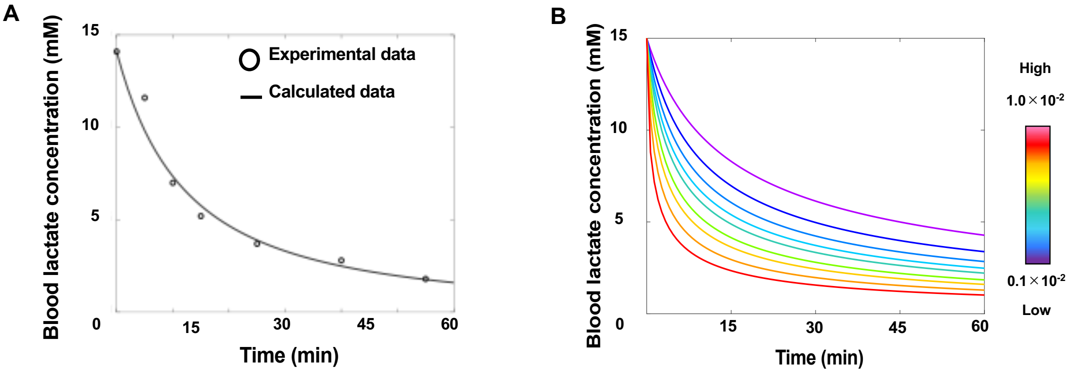

Fig. S2. Effect of 6-week endurance exercise training in the gastrocnemius (gas), soleus (sol), and plantaris (pla) muscles. The images of sol and pla are turned upside down in order to keep the order of the samples the same.

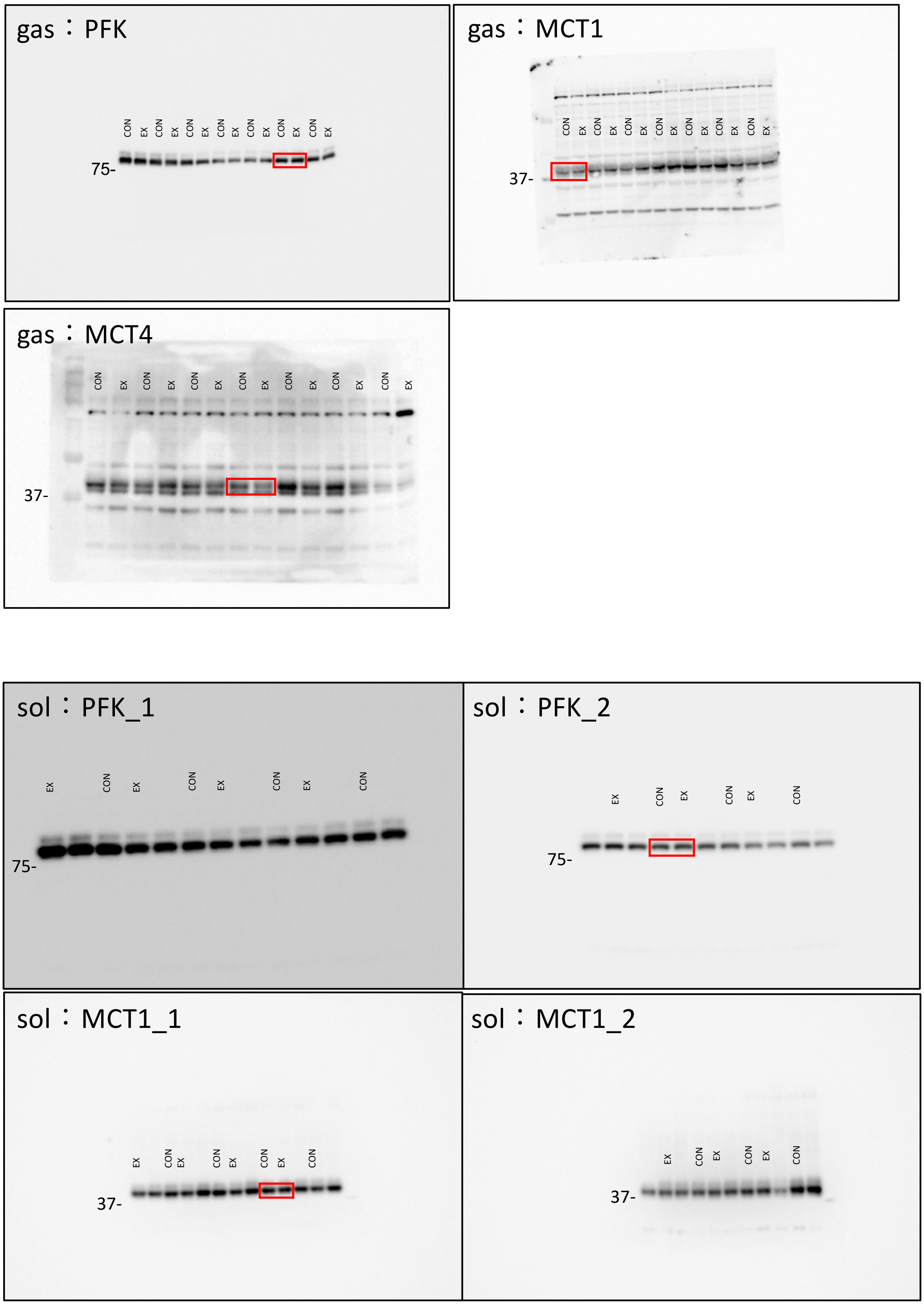

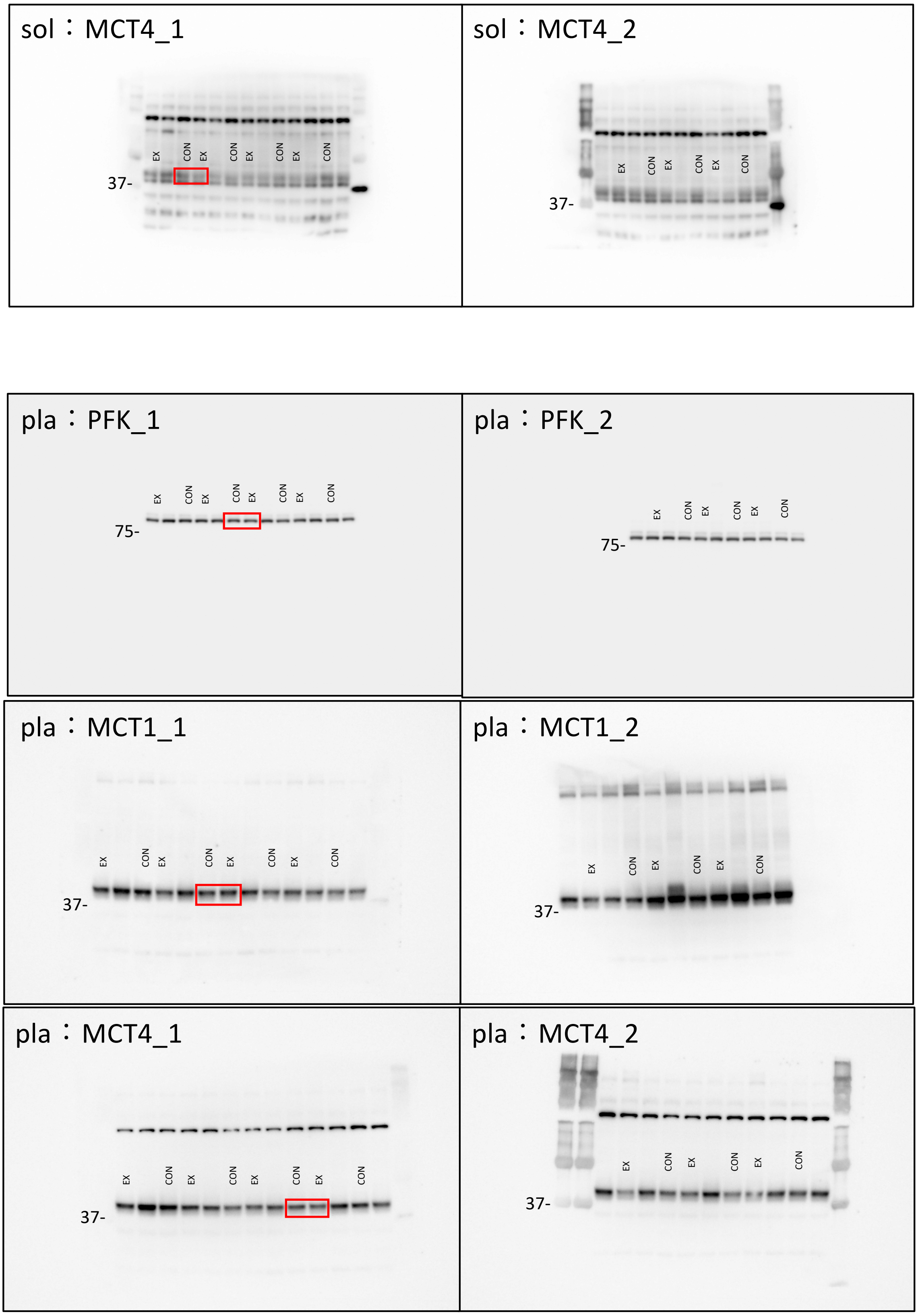

sol：MCT4_1
EX CON EX CON EX CON EX CON
37-
sol：MCT4_2
EX CON EX CON EX CON
37-
pla：PFK_1
EX CON EX CON EX CON EX CON
75-
pla：PFK_2
EX CON EX CON EX CON
75-
pla：MCT1_1
EX CON EX CON EX CON EX CON
37-
pla：MCT1_2
EX CON EX CON EX CON
37-
pla：MCT4_1
EX CON EX CON EX CON EX CON
37-
pla：MCT4_2
EX CON EX CON EX CON
37-

Fig. S3. Effect of skeletal muscle-specific PGC-1α overexpression in the gastrocnemius (gas),

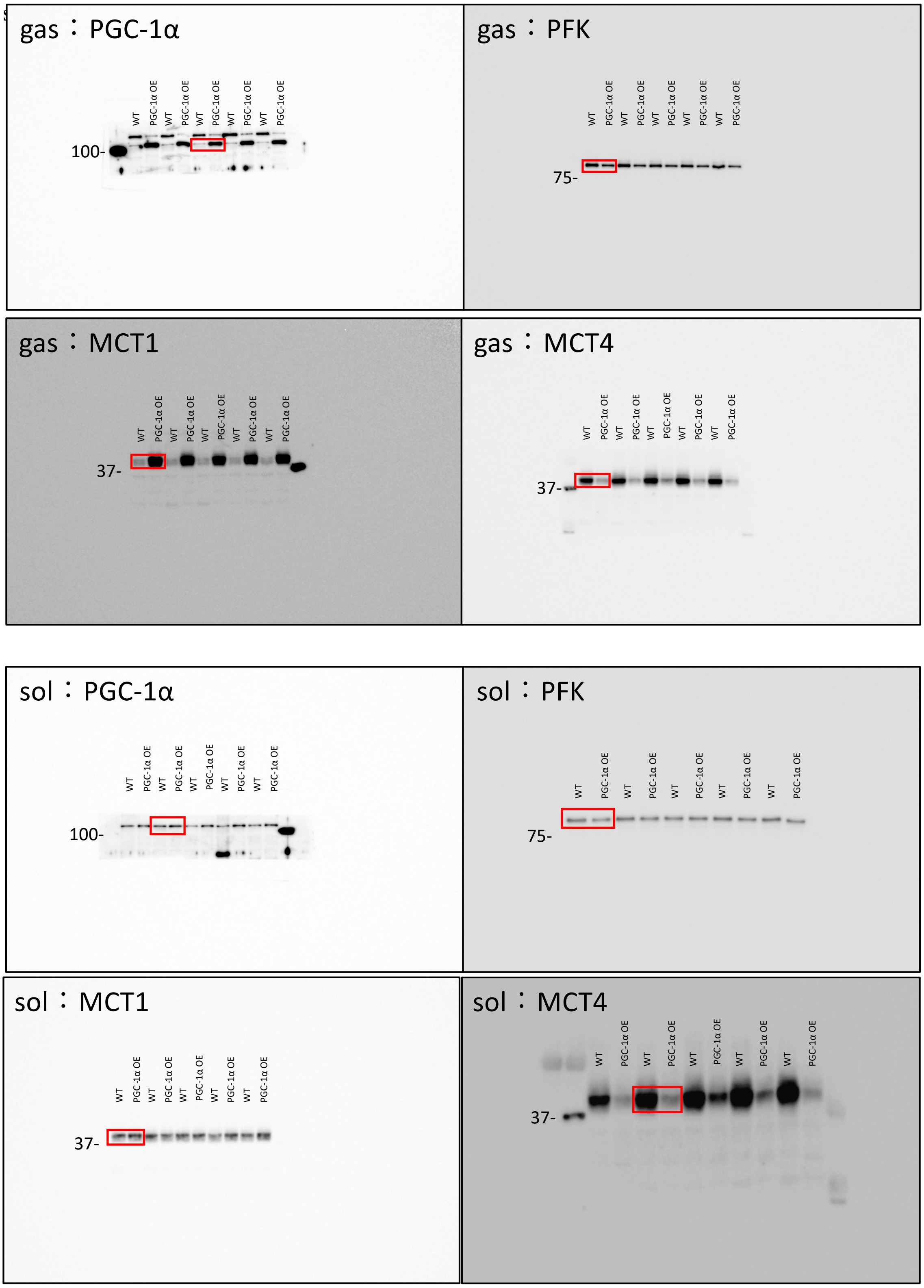

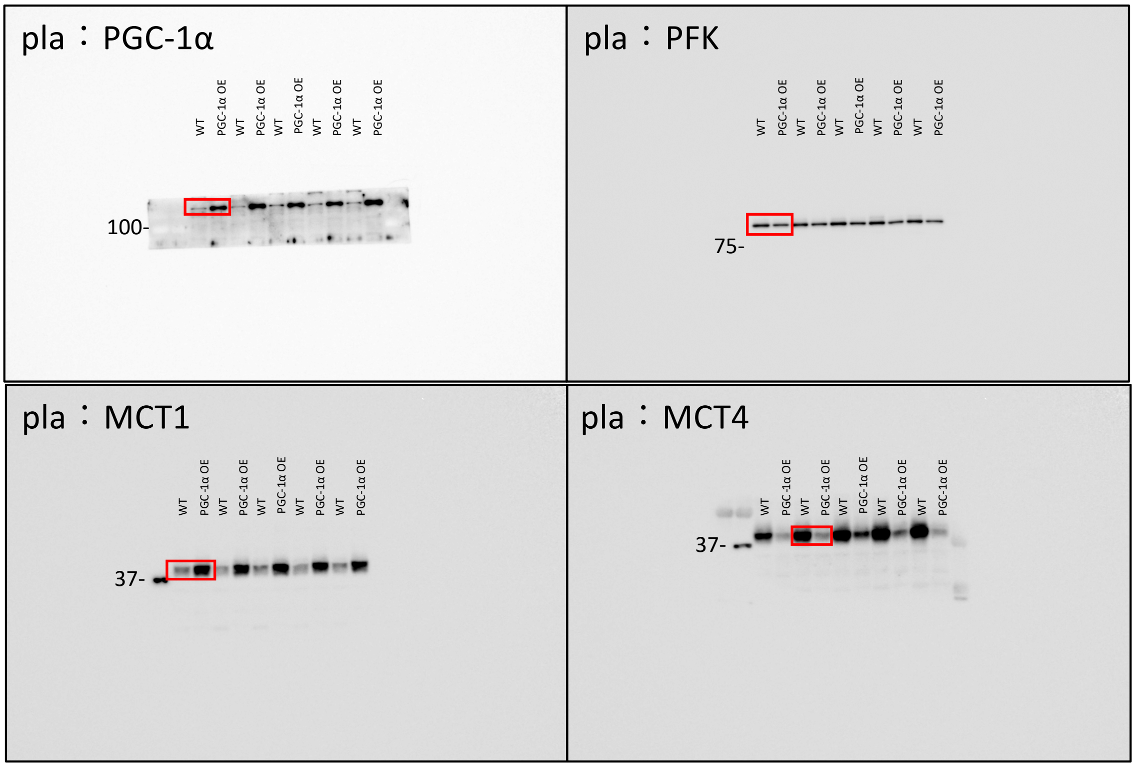
pla：PGC-1α
WT
PGC-1α OE
100-
pla：PFK
75-
pla：MCT1
37-
pla：MCT4
37-

Fig. S4. Acute response to various exercise intensities.

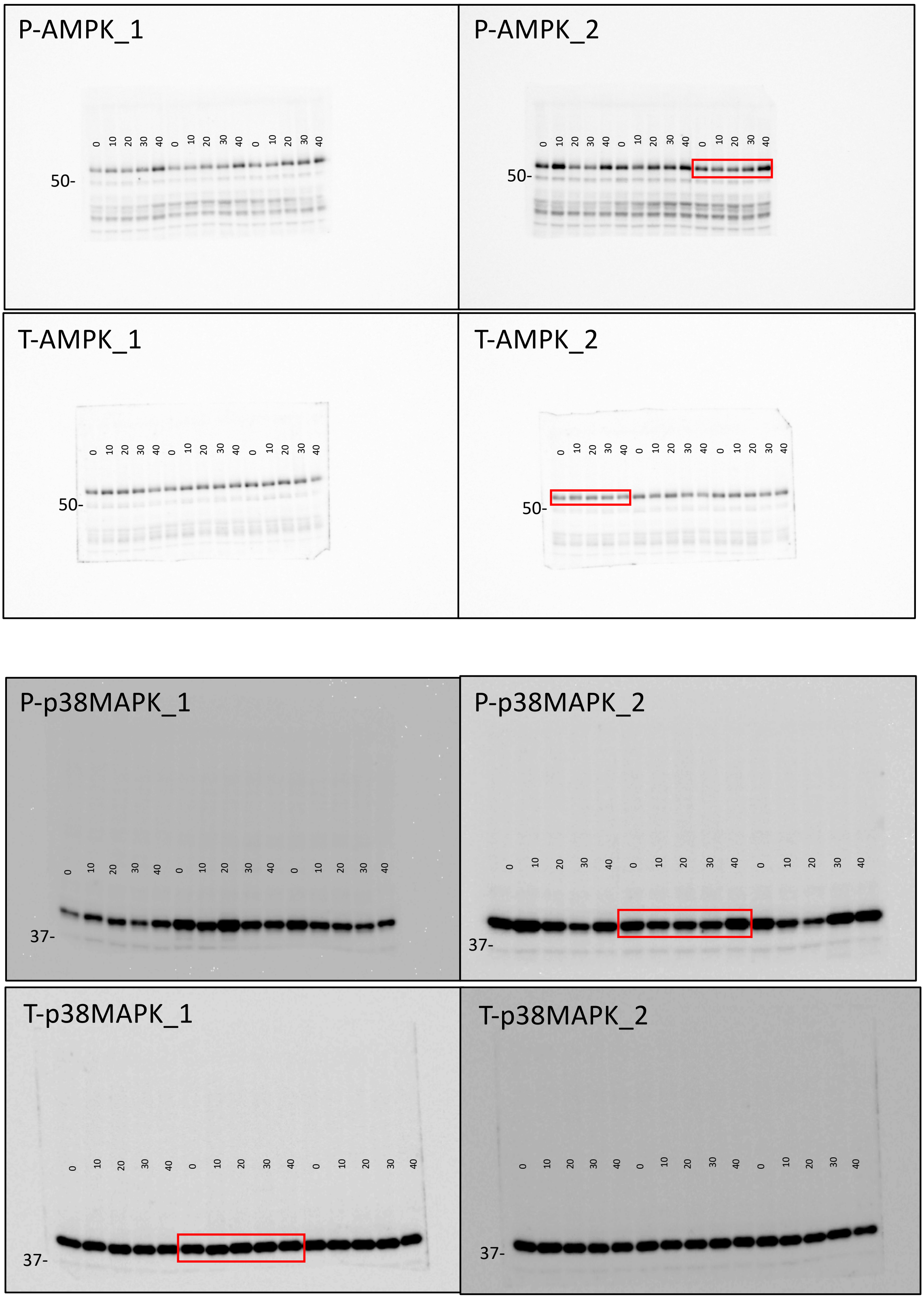

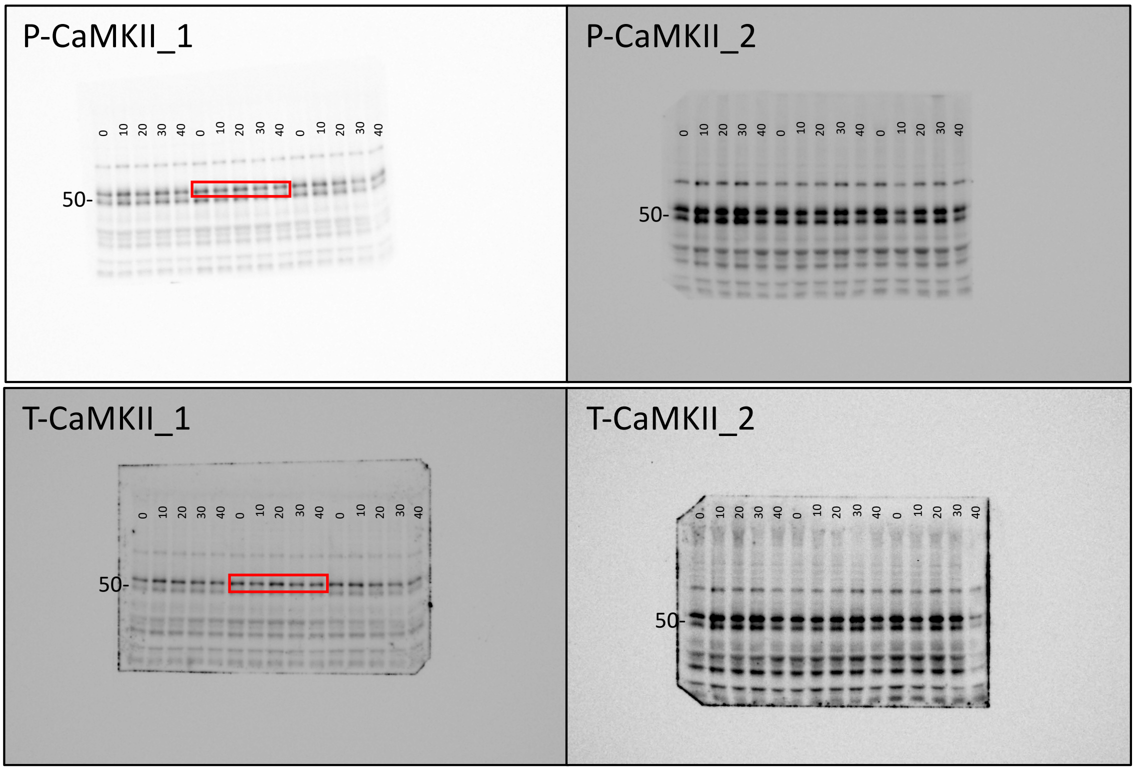
P-CaMKII_1
P-CaMKII_2
T-CaMKII_1
T-CaMKII_2
0 10 20 30 40 0 10 20 30 40 0 10 20 30 40
50-

Fig. S5. Representative Ponceau stains for each sample

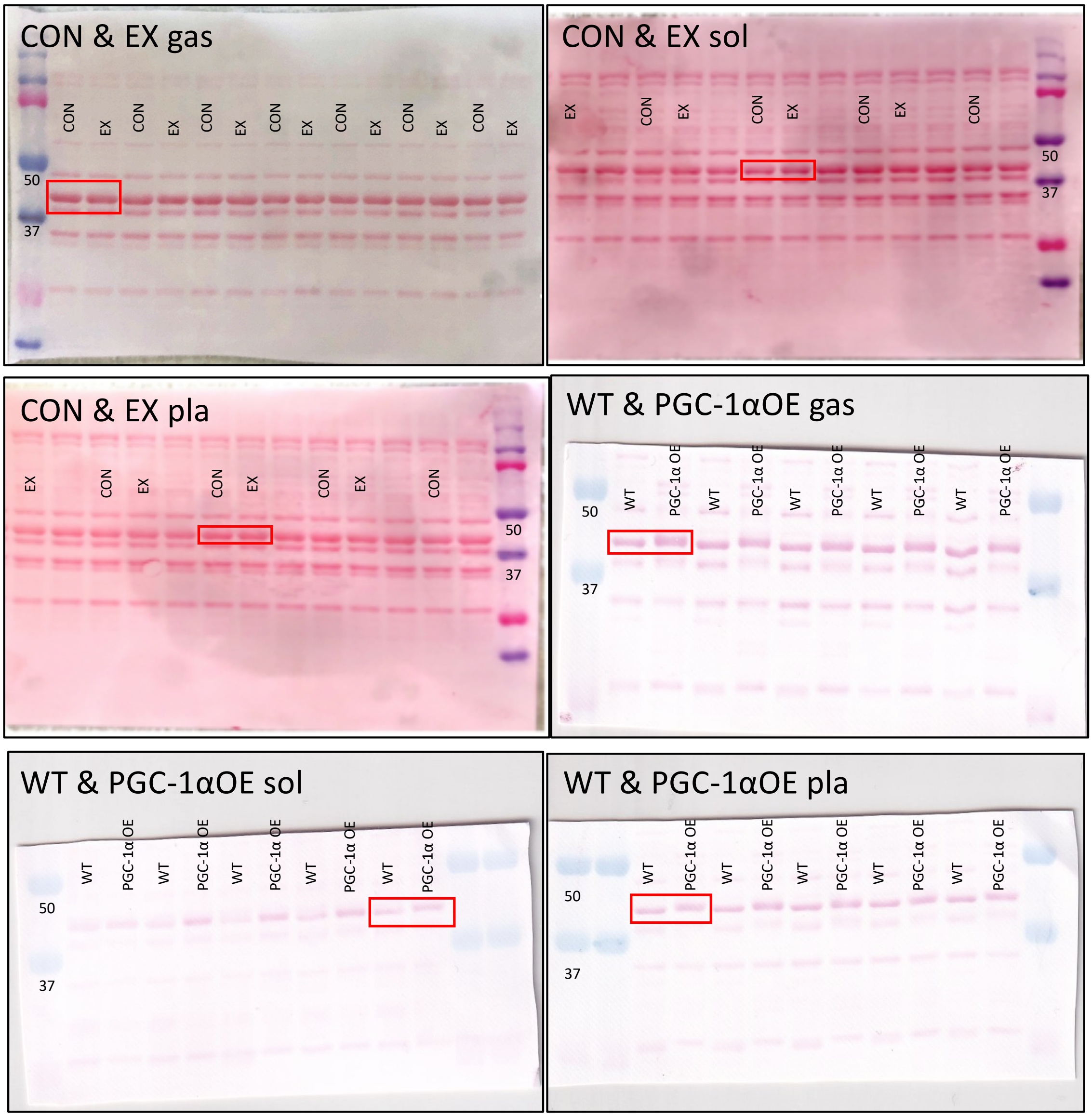

Supplement: Supplementary file 1 — Supplementary Information. [file 41598_2022_5593_MOESM1_ESM.pdf]
